# The urban footprint of rural forced displacement 

**DOI:** 10.1007/s43762-024-00148-8

**Published:** 2024-11-14

**Authors:** Edwar A. Calderon, Jorge E. Patino, Juan C. Duque, Michael Keith

**Affiliations:** 1 School of Natural and Built Environment, Queen’s University, Belfast, UK; 2 OECD Sahel and West Africa Club, Paris, France; 3 Carbon Solutions, Saint Paul, MN USA; 4 Centre on Migration, Policy and Society, University of Oxford, Oxford, UK

**Keywords:** Internally displaced persons, Socio-spatial theory, Urban marginal settlements, Housing solutions, Lefebvrian Framework, R2, R31, O18, O15, J61

## Abstract

The rapid growth of marginal settlements in the Global South, largely fueled by the resettlement of millions of internally displaced people (IDPs), underscores the urgent need for tailored housing solutions for these vulnerable populations. However, prevailing approaches have often relied on a one-size-fits-all model, overlooking the diverse socio-spatial realities of IDP communities. Drawing on a case study in Medellin, Colombia, where a significant portion of the population consists of forced migrants, this interdisciplinary study merges concepts from human geography and urban theory with computational methods in remote sensing and exploratory spatial data analysis. By integrating socio-spatial theory with quantitative analysis, we challenge the conventional housing paradigm and propose a novel framework for addressing the housing needs of IDPs. Employing a three-phase methodology rooted in Lefebvre’s theoretical framework on the production of space, including participatory mapping, urban morphology characterization, and similarity analysis, we identify distinct patterns within urban IDP settlements and advocate for culturally sensitive housing policies. Our analysis, focusing on Colombia, the country with the largest IDP population globally, reveals the limitations of standardized approaches and highlights the importance of recognizing and accommodating socio-cultural diversity in urban planning. By contesting standardized socio-spatial practices, our research aims not only to promote equality but also to foster recognition and inclusivity within marginalized communities.

## Introduction

Socio-spatial and geospatial theories have demonstrated that urban morphology and population’s sociocultural background are intrinsically related (Lefebvre, 1991; Watkins, 2005; Merrifield, 2006; Leary-Owhin, 2016; Kuffer et al., 2017; Taubenböck et al., 2018). According to Lefebvre socio-political spatial practices imprint the morphology of human settlements through three components: how people perceive space in relation to their habits and patterns of movements (spatial practice); how authorities, technocrats, and politicians intervene in the space (representations of space); and how people, individually and collectively, associate their physical and mental experiences with spatial attributes that become significant to them (representational spaces) (Lefebvre, 1991).

Recently, remote sensing and geospatial technologies have been used to measure spatial features of human settlements (Clifton et al., 2008; Taubenböck & Kraff, 2014; Kuffer et al., 2017; Angel et al., 2018; Taubenböck et al., 2018; Sharifi, 2019). These studies indicate considerable physical differences between marginal and formal urban areas in cities (Hofmann, 2001; Duque et al., 2015; Taubenböck et al., 2018), also demonstrated differences with other marginal areas from different cities (Kuffer et al., 2016; Duque et al., 2017; Taubenböck et al., 2018) and from the same city (Kuffer & Barros, 2011; Taubenböck & Kraff, 2014; Kuffer et al., 2017). This indicates that every human settlement has its own singular spatial footprint.

Our analysis focuses on marginal settlements in Colombia, country with the largest number of internal displaced populations (IDPs) worldwide (UN Refugee Agency, 2020) with more than 8.3 million to date (Unidad para las Víctimas, 2022). Those settlements emerged in the mid-twentieth century following waves of rural-urban migration attracted initially by economic prosperity, later, they became a survival refuge for forced of armed conflict. By 2015, approximately 880 million people were living in marginal settlements in developing countries (UN-Habitat, 2015). This represents a major challenge for local governments since socio-spatial studies have demonstrated that integrating different cultural populations is not a simple undertaking (Snauwaert, 2003; Berry, 2005; Sam & Berry, 2010; Ward, 1996; Ward and Kennedy,^1999^). Moreover, population’s resettlement resulting from violence, produces acculturative stress that intensifies cultural separation and marginalization.

In Medellin, the second largest Colombian city, with an urban population of 2.3 million inhabitants (DANE, 2018), approximately one in five people is an IDP (Unidad para la Víctimas, 2022). They have resettled under precarious conditions in risk-prone areas in self-build shelters forming multicultural marginal settlements due to their diverse origins. The IDPs in Medellin, once settled, start a process of collective construction of symbolic spaces of significance, such as: paths, memorials, community gardens, etc. However, this process is often interrupted with territorial interventions by the government, which impose unfamiliar ways of dwelling. This undermines the meaningful spaces and symbols the communities have built (i.e., spontaneous outdoors gathering places on sidewalks), and essentially erases the community “representational spaces.”

Despite their diverse socio-spatial-cultural backgrounds, the state established high-rise standardized apartment buildings on the outskirts of urban areas for dwelling IDPs. Flinn et al. (2017) argue how some post-disasters recovering housing programs in The Philippines have been less effective solutions due to its one-size-fits-all approaches that dismiss the variety nature of needs under resettlement circumstances. This furthers segregation, marginalization, and exclusion of these communities increasing the challenges associated with integration and access to opportunities and jobs in the formal city (Berry, 2005; Sam & Berry, 2010). This type of dwelling also appears to deteriorate socio-spatial life as it does not embrace with the inhabitants’ social space. These arguments led us to raise the following questions: Are there particular features embedded in the built environment that can help to distinguish the sociocultural backgrounds of the IDPs? If yes, how can they be self-evident to pursue more humanized housing solutions policy making?

To address these questions, we have integrated socio-spatial theory with quantitative analysis, enabling us to propose enhancements to housing solutions while challenging the established one-size-fits-all approach for IDPs. Thus, following the Lefebvrian theoretical framework on production of space (applied to geographies of displacement), we elaborate a successive three-phase research methodology: (1) participatory mapping, (2) urban morphology characterization and (3) similarity analysis. Then, we analyze the results and correlate them with Lefebvre’s socio-spatial theory to propose an innovative framework to address the design and building of housing solutions for IDPs in cities of the Global South.

## Understanding production of (informal) urbanization in geographies of displacement under Lefebvre’s socio-spatial lens

Urbanization processes in geographies of conflict (such as in Colombian cities) is usually disassociated from the local socioeconomic and cultural milieu. Moreover, contemporary authors such as Harvey (2006), Marcuse (2009) and Soja (2010), have argued the emerging spatial consciousness regarding human rights: inclusion-exclusion, right to the city, socio-spatial justice, and so on. Similarly, Parrack et al. (2017) highlighted the recent tendency to analyze conflict from spatial dimensions (i.e., land rights). For Lefebvre (1991), space itself was not the subject for analysis, rather the process of production of social space. His work, The Production of Space, has had profound influence on contemporary socio-spatial analysis. For instance, Harvey’s examination of Lefebvrian theory explicitly highlights how the physical space around us is the resulting materialization of our experiences, thus, as our experiences continuously change, space does too (Harvey, 2006). So, what does this mean within a forced displacement-resettlement scenario in informal contexts? Moreover, the unpredictable nature of the armed conflicts in Colombia has made territorial planning a complex challenge to provide a rapid and assertive response to these populations. Therefore, today, we are facing a large accumulation of informality without resolution and contradictory, enforced spatial practices, following Lefebvre’s Spatial Triad theory.

In Colombia (as in many countries in conflict in the world), the resettlement of IDPs from myriad (rural) origins in cities has highlighted a humanitarian crisis that remains unresolved. The multi-cultural “urban kaleidoscopes” resulting from these processes are viewed by authorities rather as a complex matter to be solved through a humanitarian approach. However, housing IDPs are not only a humanitarian response but also political one (Boyle, 2017). Therefore, appropriate policies with viable implementation in geographies of displacement-resettlement become necessary to respond to long-lasting housing for IDPs. What appropriate housing policies could be set if there is not a deep understanding of the socio-spatial phenomena in these territories? How is it possible to overcome the long-lasting homogenizing response to IDPs housing? Examining Lefebvrian socio-spatial theory, specifically his Spatial Triad conceptualization, could provide insights into how to undertake this endeavor.

The common unpredictable nature of conflict blended with the accelerated rates of forced displacement around the world have contributed significantly to the formation of marginal settlements in the periphery of the cities of the Global South. These have created new forms of forced urbanization that are often not officially recognized due to their lack of formality (Calderon, 2021). In these marginalized territories, conflict is fueled and shaped by relations of power that creates a rupture between the discourse and praxis associated with space. This manuscript focuses on that rupture considering Lefebvrian Spatial Triad. How the Spatial practice (how space is perceived) and Representational spaces (how the space is lived) clash with Representations of space (how space is conceived). In other words, the struggles IDPs face when spatial planning authorities (e.g., planners, policy makers, architects) overlook the way in which they inhabit, relate to, and experience the built environment resulting in unjust enforced dwelling solutions. For Lefebvre (1991, pp 43–44), “the producers of space have always acted in accordance with a representation, while the users passively experienced whatever was imposed upon them inasmuch as it was more or less thoroughly inserted into, or justified by, their representational space.”

IDPs resettlement has been discussed in recent decades as one of the worldwide humanitarian priorities by international agencies (e.g., UN Refugee Agency, International Organization for Migration), academics from various disciplines, and cross-sectoral stakeholders such as the National Centre of Historical Memory in Colombia, which has exposed the impact in the urban configurations of cities by massive resettlement of IDPs (Centro Nacional de Memoria Historica, 2015). In the late 1990s, Michael Cernea, as part of The World Bank, undertook a comprehensive study regarding the socioeconomic implications of displacement for the evicted populations such as landlessness, homelessness, and social disarticulation (Cernea, 1997). For the latter, he proposed the resettlement of displaced populations in “groups and social units” to maintain social ties. This proposal seems to rarely apply to geographies of violent conflict where displacement and resettlement is out of control. However, Cernea’s point is crucial and still relevant for more recent studies related to the importance of social ties for the resettlement process. This is one of the main arguments of this manuscript. From a policy perspective, Escobar discusses displacement as the result of capitalist modernity and points the need for revising legal mechanisms that protects IDPs “resettlement [which] should be seen as the exception, not the rule, and as a temporary, never permanent measure” (Escobar, 2003, pp. 164).

Discussion of IDPs housing has taken many forms and draws upon a variety of perspectives such as practices of self-recovery, contesting the one-size-fits-all approach discussed by Flinn et al. (2017). The latter was censured by Lefebvre (1991) as a multi-dimensional homogenizing tendency of humanity that reproduces the dominant economic structure of the global market. Other authors address the dwelling recovery as a process of community empowerment (Wain, 2017; Stephenson, 2018) that must include users (IDPs), local built techniques, and an interdisciplinary data-driven design for comprehensive shelter solutions (Terne et al., 2017; Goddeeris & McDonald, 2017). These approaches have been in Lefebvrian terms, an attempt to overcome “the dominated- and hence passively experienced|” space (Lefebvre, 1991, pp. 39), endured by IDPs in their everyday life through the hegemonic commodification of space.

In this manuscript, through empirical interdisciplinary research we explore the production of space in informal settlements that have emerged because of the resettlement of multicultural origin IDPs. Lefebvre suggested that what differs representations of space and representational spaces is culture, which materializes in distinct conceptions of socio-spatial realities (Lefebvre, 1991). Furthermore, for Lefebvre, the authoritarian rationalization of social space is dominated by abstraction that in contexts of violent conflict is political and when it is established by the state it is institutional (1991). Therefore, this rationalization translates into a logic of efficiency and standardization that have been the gatekeepers for urban planning policies in cities such as Medellin (Calderon, 2017), where socio-spatial heterogeneity is not recognized. Rather homogenized through low-income mass-housing developments that neglect IDPs’ culture, traditions, and socio-spatial practices (Calderon, 2021). Contesting standardization socio-spatial practices struggle to achieve equality, rather to gain recognition: ultimately equality homogenizes, but recognition diversifies.

## New cartographies of displacement: raising awareness combining socio-spatial and geospatial methods

The magnitude and unpredictable nature of forced displacement have challenged the infrastructure and technical capability of local governments to monitor the rapid emergence of marginal settlements. In 2020, the UN Refugee Agency (2020) reported that every two seconds someone in the world is displaced from their home, so, how local governments could cope with an overwhelming situation like this?

Combining qualitative and quantitative methods, we documented the informal land occupation by a rural diaspora of IDPs in Medellin and contributed with theoretical, practical, and methodological elements to strengthen local capacities to respond more assertively to forced displacement. While in theory, Lefebvre´s socio-spatial work is analyzed in the context of conflict exposing empirical evidence, in praxis, it provides an operational tool that can help policymakers and local authorities to design strategies to more appropriately house IDPs. However, we argue the imperative collaboration across disciplines to seek innovative housing solutions using quantitative and qualitative methods for the integration of IDPs to the larger society in Colombian cities (and likely in many cities worldwide).

### Participatory mapping

**Fig. 1:**
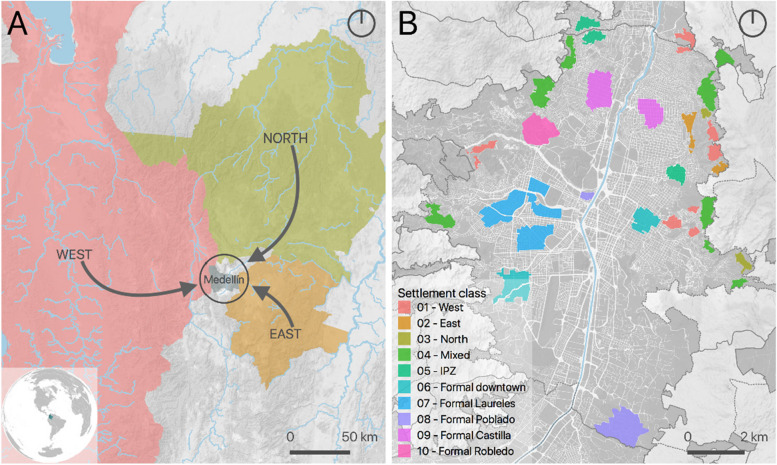
Settlements and origins. **A** Origins of IDPs living in Medellin. **B** Location of analyzed settlements (formal and marginal). Marginal settlements are named after the main origin of their populations, for pragmatic reasons

We gathered two separate groups of experts to map the city’s marginal settlements establishing the main origin of their populations. Group A, academics and public officers who have been working in marginal settlements in Medellin, and group B, community members, including IDPs, who live in, and have extensive knowledge of the settlements. We asked both groups to (1) delineate the settlements over a high spatial resolution image of the city and (2) identify, for each settlement, the main geographical origin of its population, its estimated date of emergence, and the main cause of the displacement. We integrated the gathered information from both groups into a single map and then used a regular grid to extract quantitative information from different geospatial datasets to characterize the physical urban morphology of each class of settlement (see Fig. 1). For comparison purposes, we also include a consolidated area that emerged as an informal settlement some decades ago that we call the informal planned zone (IPZ) and five formal areas of the city that were properly planned.

### Urban morphology characterization

After the participatory mapping, we created a 100-meter-wide cells hexagonal grid of the area of Medellin and selected the cells that are located within the identified marginal settlements. Then we extracted image and terrain metrics from a very high spatial resolution orthophoto, a Landsat 8 image, and a digital elevation model (DEM). This dataset contains 1,595 records (cells) with polygon geometry, stored in an ESRI shapefile format using WGS 84 (EPSG:4326) as the coordinate reference system (MDE_settlement_class_metrics.shp). Each record contains information on the settlement class and 31 morphology metrics extracted from the geospatial datasets. We used a very high spatial resolution (VHR) orthophoto of the city from 2016 (50 cm pixel size) to extract image features, a Landsat 8 image from 18 December 2017 to extract spectral indexes, and a digital elevation model (DEM) to extract terrain descriptors.

For image feature extraction we used an automatic software tool called FETEX 2.0 (Ruiz et al., 2011) to compute the image metrics from the orthophoto (FETEX 2.0 is available at the Geo-Environmental Cartography and Remote Sensing Research Group website: http://cgat.webs.upv.es/software/). Image metrics are measurements that we can extract from aerial or satellite imagery that quantify specific aspects of the urban morphology, such as descriptors of the land cover colors (spectral metrics), the image texture (texture metrics), or the spatial arrangement of the different objects in the image (structure metrics). Other metrics can be extracted by using spectral indices that relate directly to the presence of certain features in each place, such as vegetation, through the normalized difference vegetation index (NDVI), or the normalized difference built-up index (NDBI) that quantifies the presence of built-up surfaces.

These metrics allow an automated, standardized characterization of the urban morphology, and they can be used in any urban area in the world using aerial or satellite imagery. These same image metrics have been previously used to map informal areas from VHR images in Latin American cities with good performance (Duque et al., 2017). We used map algebra to calculate the Landsat spectral indexes, and zonal statistics to calculate the terrain descriptors from the DEM (see Table 1, where the image metrics are grouped into spectral metrics, texture metrics, and structure metrics).

The spectral metrics provide information about the color of the image pixels, which reflect land cover features (e.g., roof types, vegetation). In this case, we recorded the mean and standard deviation values on each of the three image bands for a total of six metrics in the group. Structural and texture metrics are abstract measures that allow to quantitatively differentiate organic, crowded, and cluttered spatial patterns from the more structured, ordered and homogeneous urban layouts typical of wealthy neighborhoods (Duque et al., 2015). The texture metrics provide information about image contrast, uniformity or homogeneity, and rugosity, and the group contains 11 different image texture descriptors. The structure metrics characterize the randomness or regularity of the spatial distribution of the elements, thus providing more information about the spatial pattern and complementing the information provided by the texture metrics. This group contains ten different metrics. For more information on the definition and calculation of the image metrics, see Ruiz et al. (2011). The Landsat image was used to calculate two spectral indexes, namely, the previously mentioned NDVI and NDBI.

**Table 1:** Morphology metrics extracted from geospatial datasets. Image metrics from the 2016 Orthophoto, spectral indexes from the 2017 Landsat 8 image, and terrain metrics from the DEM. For a detailed explanation of the image metrics, i.e., spectral, texture, and structure metrics, please see Ruiz et al. (2011)

Group	Metric	Description
Spectral metrics	MEAN1	Mean value of the red band.
	DEVST1	Standard deviation of the red band.
	MEAN2	Mean value of the green band.
	DEVST2	Standard deviation of the green band.
	MEAN3	Mean value of the blue band.
	DEVST3	Standard deviation of the blue band.
Texture metrics	MEAN_EDG	Mean of the edgeness factor.
	DEVST_EDG	Standard deviation of the edgeness factor.
	UNIFOR	Gray level co-occurrence matrix uniformity.
	ENTROP	Gray level co-occurrence matrix entropy.
	CONTRAS	Gray level co-occurrence matrix contrast.
	IDM	Gray level co-occurrence matrix inverse difference moment.
	VARIAN	Gray level co-occurrence matrix variance.
	CORRELAC	Gray level co-occurrence matrix correlation.
	SKEWNESS	Skewness value of the image histogram.
	KURTOSIS	Kurtosis value of the image histogram.
Structure metrics	RVF	Ratio variance at first semivariogram’s lag.
	RSF	Ratio between semivariance values at second and first semivariogram’s lags.
	FDO	First derivative near the origin of the semivariogram.
	SDT	Second derivative at third semivariogram’s lag.
	MFM	Mean of the semivariogram values up to the first maximum.
	VFM	Variance of semivariogram values up to the first maximum.
	DMF	Difference between MFM and the semivariance at first lag.
	RMM	Ratio between the semivariance at first local maximum and the mean semivariogram values up to this maximum.
	SDF	Second order difference between first lag and first maximum.
	AFM	Area between the semivariogram value in the first lag and the semivariogram function until the first maximum.
Spectral indexes	NDVI_mean	Mean of the normalized difference vegetation index.
	NDBI_mean	Mean of the normalized difference built-up index.
Terrain metrics	Height_mean	Mean height above sea level.
	Slope_mean	Mean slope.

**Fig. 2:**
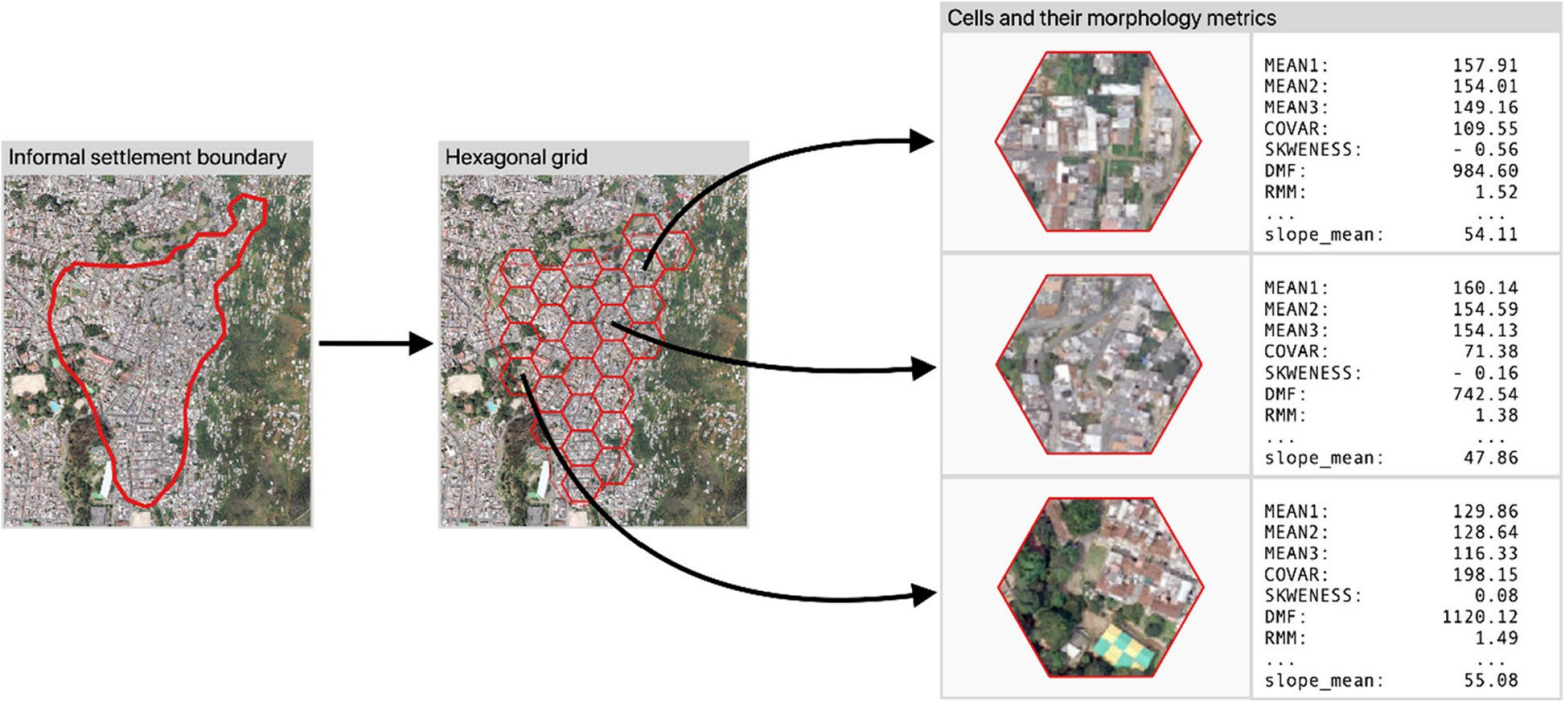
Example of the extraction process of urban morphology metrics (with the corresponding values) from three different hexagonal cells from an informal settlement

We used the DEM to quantify terrain height (mean height) and slope (mean slope). A conventional zonal statistics tool was used to extract the mean NDVI, mean NDBI, mean height and mean slope for each hexagonal cell in the dataset. Figure 2 shows an informal settlement and some of the extracted metrics for three different hexagonal cells.

The integration of cultural and physical characteristics is central to our methodology and analysis. The participatory mapping phase captures the socio-cultural practices of displaced communities, while the urban morphology characterization quantifies the physical adaptations in settlement patterns. These two dimensions are deeply interconnected. For example, many IDPs adapt their homes and settlement structures to both the terrain and their cultural practices from their regions of origin. The construction methods and spatial organization observed in these settlements (such as the use of stilts, terraces, or organic layouts following natural topography) are not just responses to physical constraints but also reflect deeply ingrained cultural practices. By linking these socio-spatial practices with morphological metrics, we underscore the importance of considering both cultural and physical factors in designing housing solutions for IDPs.

The physical environment of IDP settlements in Medellín is deeply influenced by the cultural backgrounds and socio-economic conditions of displaced communities. IDPs, having been displaced from rural areas, bring with them a range of socio-cultural practices that shape the way they adapt to the urban landscape. These practices, rooted in rural traditions, are not only about survival but also about the reconstruction of their social and physical spaces in the absence of institutional support. The resettlement of IDPs in marginal and risk-prone areas forces communities to rely on self-cooperative approaches that reflect a blend of resilience, resourcefulness, and cultural heritage.

Contrary to the assumption that these settlements are entirely unplanned or chaotic, many are informally organized with strategic planning led by community leaders or pirate developers. As noted by Dovey and King (2011), land invasions are often accompanied by “formal” street plans and lot layouts, and IDPs invest significant time and resources in developing communal infrastructure. In Medellín, IDPs have collectively built community walkways, staircases, roads, aqueducts, and sewer systems, as well as constructed their own homes, recreational spaces, and urban art, all of which reflect their ability to adapt and innovate in challenging environments. These efforts showcase not only the physical transformation of space but also the cultural imprint of IDP communities on the urban fabric.

**Fig. 3:**
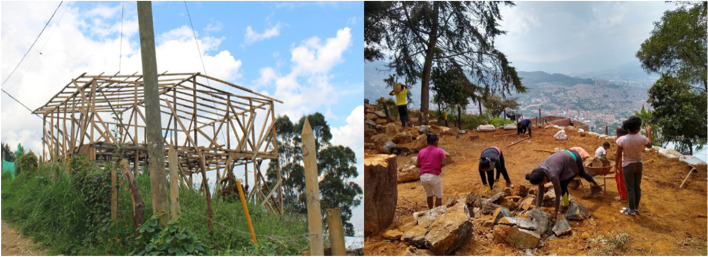
Examples of IDP settlement adaptations to challenging topographical conditions. Source: Photo taken by one of the authors

Cultural practices from rural life, such as community cooperation and collective problem-solving, are evident in the way IDPs construct their homes and neighborhoods. For example, IDPs have adapted their housing to the steep topography by employing traditional construction techniques from their regions of origin, such as building on stilts or excavating the land to create flat surfaces. These techniques are a direct reflection of the socio-cultural knowledge carried from rural settings and are shaped by the need to adjust to difficult urban geographies (see Fig. 3).

**Fig. 4:**
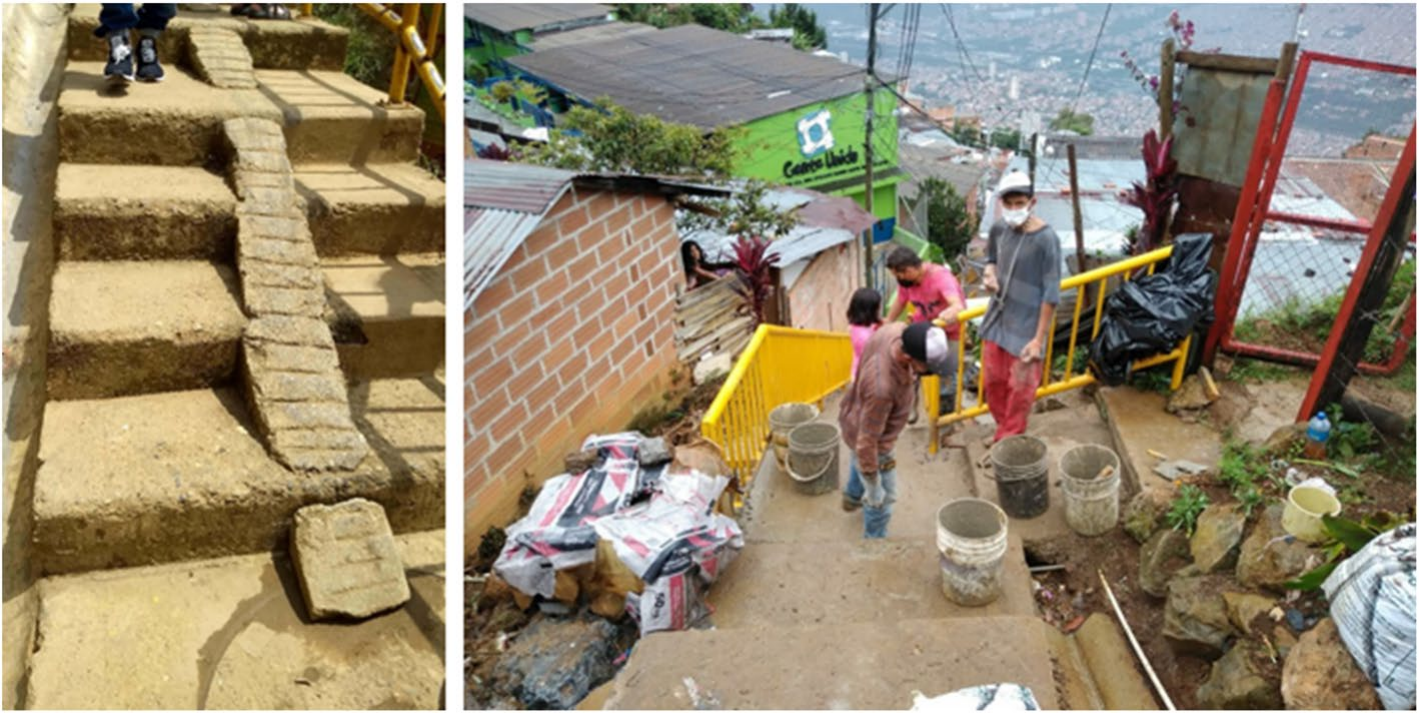
Communally built steps with ramps for motorbike access in IDP settlements. Source: Photo taken by one of the authors

Another prominent example of cultural adaptation in the physical environment is the construction of steps with ramps to allow easier access for motorbikes, a common mode of transport in these settlements. These ramps, built collectively, reflect the community’s problem-solving capabilities and their ability to adapt to local mobility challenges in steep terrains (see Fig. 4). Public infrastructure, such as roads and aqueducts, is built through communal efforts, often involving collective labor where different members contribute in various ways (some cook communal meals while others work on construction projects). This cooperative spirit is deeply rooted in the cultural fabric of rural life and continues to shape the social and physical spaces in urban IDP settlements.

The socio-economic activities of IDPs also leave a mark on the built environment. For instance, small entrepreneurial ventures such as corner shops and street food stalls not only provide livelihoods for displaced individuals but also act as important social spaces within the community. These spaces, in Lefebvrian terms, serve as “representational spaces” where daily interactions and community bonding occur, transforming physical locations into culturally significant hubs.

The integration of cultural practices with physical adaptations is essential to understanding the socio-spatial dynamics of these settlements. While financial constraints limit the materials and resources available to IDPs, their settlements are shaped by a gradual process of improvement, with homes and community spaces reflecting both the limitations and the ingenuity of the inhabitants. As such, the cultural backgrounds of IDPs play a pivotal role in influencing how physical spaces are organized and utilized in their resettlement areas.

### Similarity analysis

We then quantitatively compare and analyze the similarity among those settlements and the formal areas of the city using morphology metrics. This procedure is based on the following two premises: (1) the physical appearance of an urban settlement is a cultural reflection of the community that built it, and (2) people living in urban areas with similar physical housing conditions have common social and demographic characteristics (Duque et al., 2015; Jain, 2008; Taubenböck et al., 2009). We performed a similarity analysis between settlement classes by comparing the statistical distributions of their morphology metrics. This method to compare the urban morphology of different settlements allows us to identify the degree of compatibility of their respective populations.

We first used boxplots and parallel plots to quantitatively (visually) compare the settlement classes in terms of their metrics. Next, for each of the 30 metrics (i.e., MEAN1, MEAN2… Slope_mean), we compute the Pearson’s Correlation Coefficient between each pair of settlement classes (i.e., West, East, North, … Formal Robledo). Finally, we calculate the average between the 30 correlations available for each pair of settlement classes and present those results in a Similarity Matrix. An average correlation value close to 1 indicates a high level of similarity between the urban morphologies of two settlement classes.

**Fig. 5:**
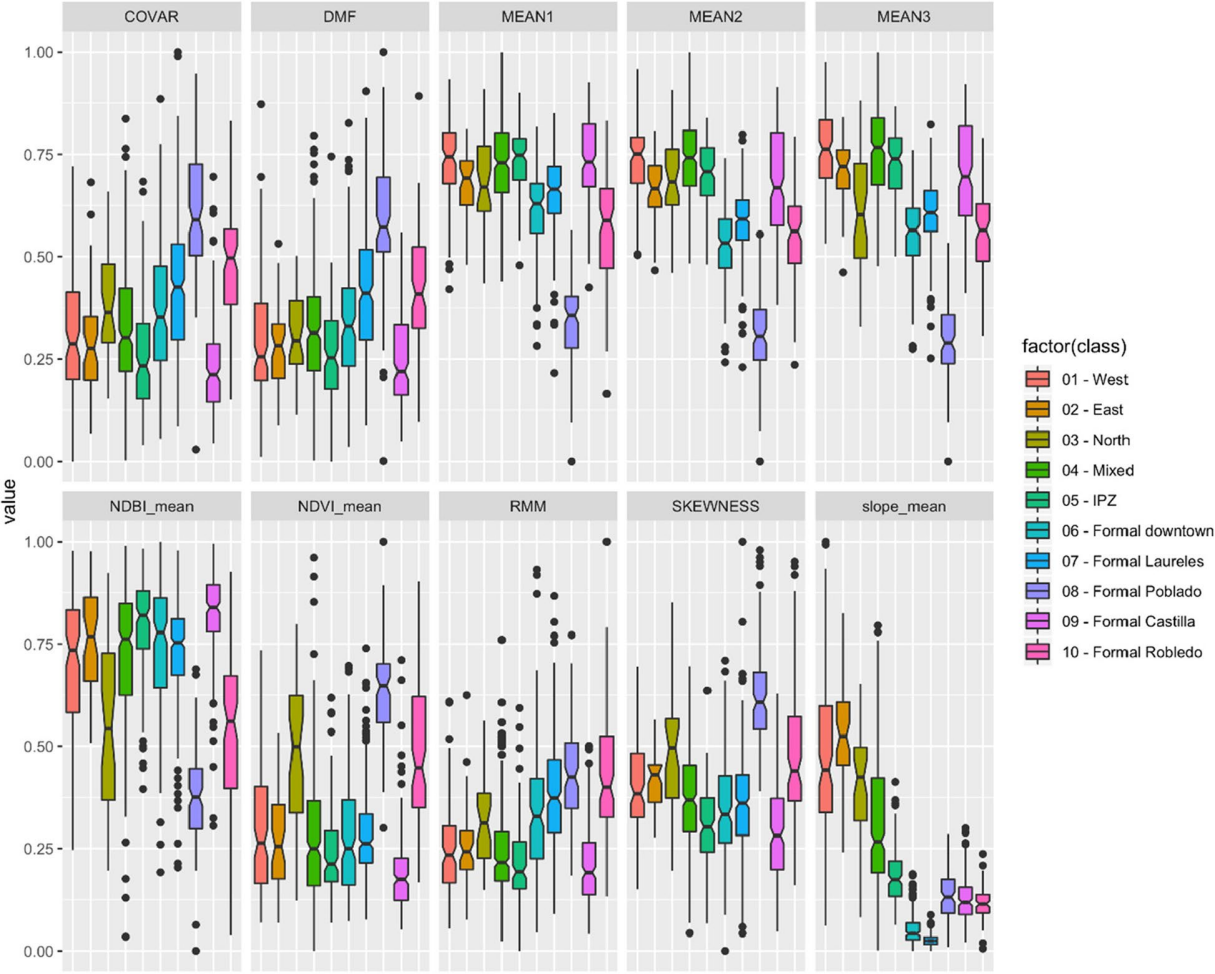
Boxplots of selected variables for each settlement class. They show the differences in the statistical distributions of values of each morphology metric

The comparison of the morphology metrics reveals differences between the urban morphology of marginal and formal areas and between marginal areas with populations from different geographical origins. Figure 5 displays values of differences among the marginal settlements. They also show that marginal and formal areas have different distributions and that the area of formal Poblado differs substantially from all of them together. NDVI and NDBI values show that the formal Poblado area is the greenest settlement, while formal Castilla is the opposite: the most built-up. The texture and structure metrics (COVAR, DMF, RMM, and SKEWNESS) show that formal Castilla has the most orderly layout. Noteworthy, the formal settlement classes show lower within-class terrain slope variability, these low values indicate mostly flat terrain. On the other hand, informal settlements show low variability in terms of color (MEAN1 and MEAN2) and structure (DMF and RMM), indicating more organic layouts and some similarity regarding the built-up covers.

**Fig. 6:**
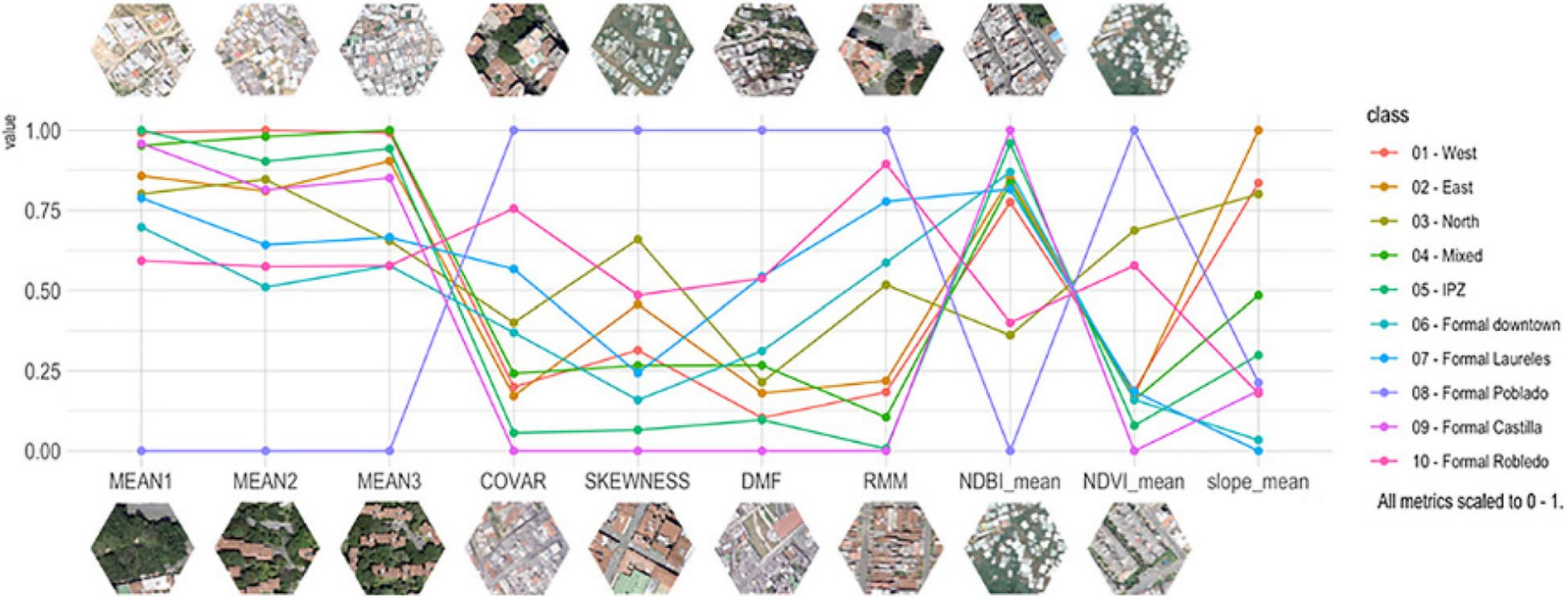
Parallel coordinates plot of selected variables for each settlement class. The plots were built using the class means for each variable to show how the central trends change among settlement classes

Figure 6 allows us a clearer visualization to identify the central trends and differences of each settlement group. It is important to note that topographic adaptation is intrinsically related to socioeconomic capacity. The wealthier area, Poblado, located in the southeast hillside zone of the city, displays the most dissimilar attributes of the settlement classes. It differs from the other formal areas and the marginal hillside settlements in the amount of vegetation and green spaces (albeit most of them are private), the occupation of land by buildings, and the layout of the streets. Poblado area consists primarily of high-rise buildings in gated communities that do not adapt to the topography as the marginal settlements do, rather, modify it. This discloses dissimilar spatial practice dynamics (in Lefebvrian terms) between the formal and informal settlements in the hillsides. The dissimilar terrain-adapted morphology follows contrasting technical and financial capacities to modify their terrain. Thus, marginal settlements reproduce socio-spatial patterns adapted to the existing topographic conditions with individual or little terrain modifications.

The fieldwork and VHR orthophoto validate the dissimilar technical and financial capacities when comparing the streets of Poblado with marginal settlements in hillsides. This materializes in divergent spatial practices determined by socioeconomic conditions and day-to-day socio-spatial patterns of movement. In Poblado, streets reach every single gated community despite its steep topography (private car is the predominant mode of transport), while in marginal settlements, very steep housing is reached by self-built steps or improvised ramps, also showing the emergence of vernacular representations of space that work as practical solutions to a socio-spatial issue (Carp, 2008). In marginal settlements the street width is inversely proportional to the degree of steepness: the steeper the gradient, the narrower the streets.

**Fig. 7:**
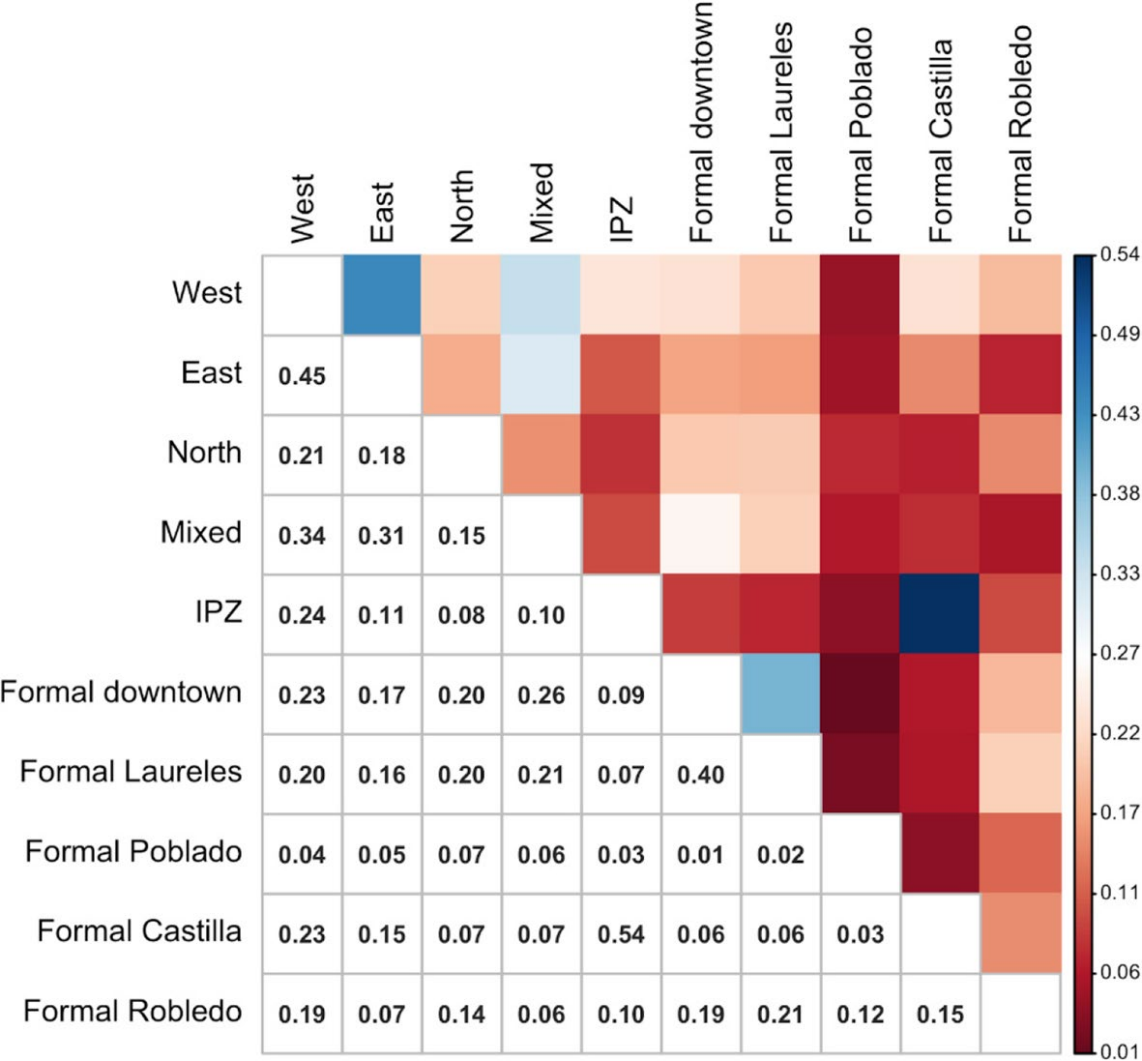
Similarity matrix. It was built using the average Pearson’s correlation coefficient between each pair of settlement classes

While urban morphology of marginal settlements looks similar at first glance, the results of the similarity analysis indicate specific levels of resemblance between settlement classes with respect to their image and terrain metrics (Fig. 7). The resulting matrix revealed that the highest similarity exists between the IPZ and formal Castilla groups, followed by west and east groups, and then between formal Laureles and formal downtown. The lowest similarity measures consistently involve the formal Poblado. The marginal settlement classes that are more alike are the east, west, and mixed groups, with the north class being more dissimilar from them. Last, the formal settlements closer in appearance to the marginal settlement classes are Downtown and Laureles.

**Fig. 8:**
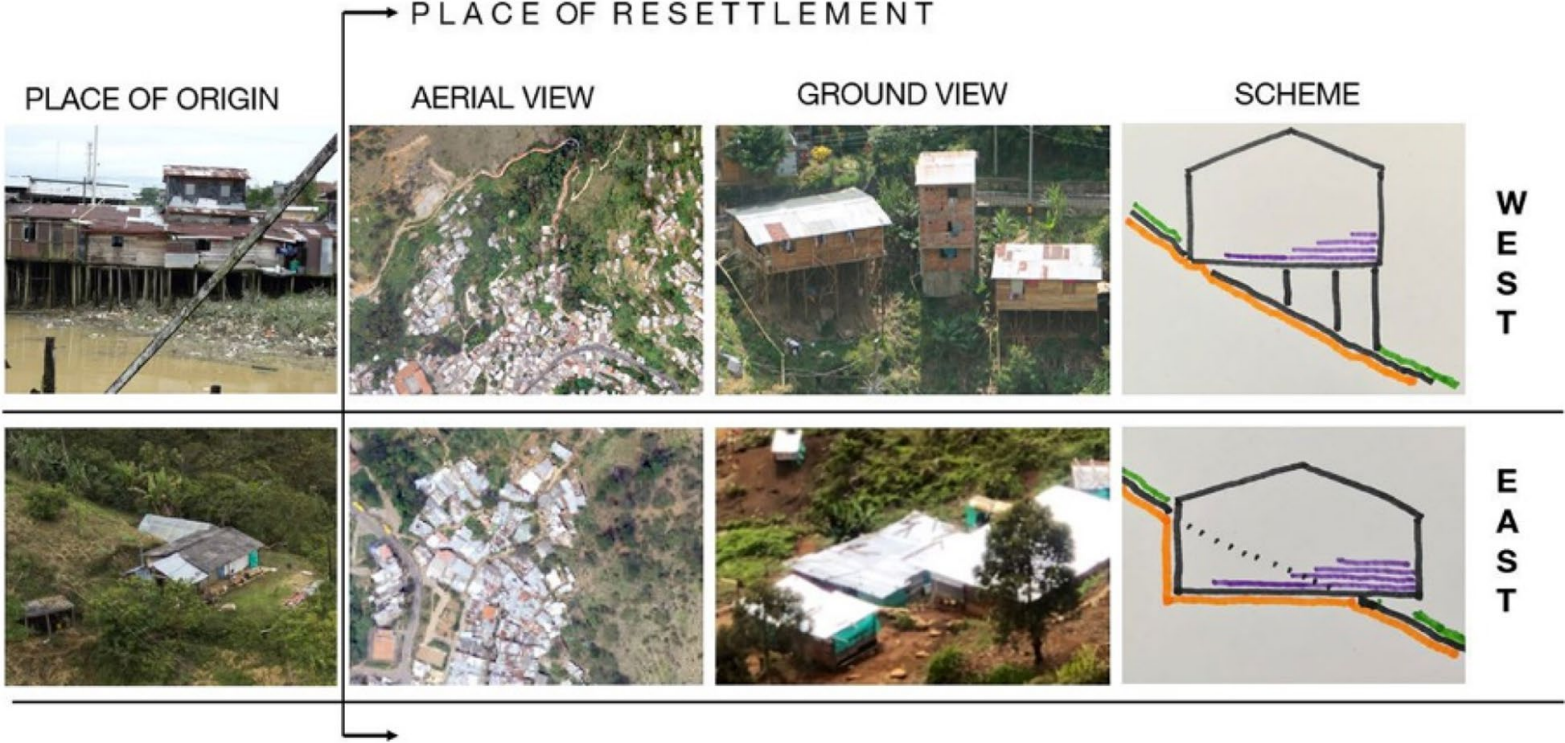
Different topographic adaptations (stilts and cut ins) related to spatial practices of IDPs from origin (west and east) to places of resettlement

The gathered evidence suggests that:

- IDPs’ cultural backgrounds create singular territorial signatures: We used several geospatial datasets to quantitatively characterize the urban morphology of both marginal and formal settlements according to their physical appearance (from aerial views and satellite imagery) and their terrain characteristics. The similarity analysis indicates that each settlement has its own signature in terms of color, texture, spatial patterns, vegetation presence, built-up density, and terrain slope (Figs. 5 and 6) and that some settlements are more similar than others (Fig. 7). These territorial footprints represent, in Lefebvrian terms, representational spaces that have been produced by IDPs’ diverse socio-spatial realities through day-to-day interactions. They are places of collective significance both intangible (i.e., symbols and experiences) and tangible (i.e., memorials and neighborhood centralities).
- IDPs’ socio-spatial backgrounds are transferred to the newly built environment: The different self-constructed housing techniques (spatial practices) identified in marginal settlements are often related to the populations’ socio-spatial backgrounds (places of origin). IDPs adapt to new living settings differently. For instance, some IDP communities adapt their houses to the terrain by erecting them on stilts, others, flattening the land using cut-ins (Fig. 8). These characteristics cannot be documented from aerial views. In addition, their similar morphological adaptations to the topography include building housing in rows following organic lines (elevation contour lines). This collective adaptation to geomorphological conditions mirrors the spatial characteristics in which IDPs lived prior to their forced displacement. These communities began their territorial colonization by building precarious shelters with affordable materials, such as timber, cardboard, plastic and tin. Then, when economic situations allow, they progressively build their houses using more stable materials, such as brick and concrete. Thus, topography is related to the use of permanent materials for construction. The steeper the gradient is, the more temporal are the materials observed, except for the formal Poblado area. This finding obeys the land occupation process of forced displaced populations, as the lower zones of the hills are already occupied and consolidated, thus newer settlers have to locate themselves in higher and steeper land.

- Resettlement of IDPs could be possible in consolidated residential areas of the city: The similarity analysis reveals similar spatial attributes among some marginal settlements also with some of the formal residential areas of the city (IPZ, Castilla, Downtown and Laureles). We acknowledge the significant challenges (i.e. discrimination, conflict), that the acculturation process of IDPs to some formal areas of the city (i.e., Downtown and Laureles) would bring due to the existing sociocultural prejudices in Colombian society towards lower-class populations. However, some consolidated formal areas such Castilla, that was planned as a working-class neighborhood, emerged from rural-urban migration attracted by the strong industrialization of Medellin in the mid twenty-century. This not only portrays a potential zone for IDPs resettlement but also appears to explain the highest similarity that our study found between Castilla and IPZ.

The IPZ are transition zones that we identified in our analysis. They represent a disconnection between the planned zones of the city with marginal settlements. These IPZs, we believe, have also potential for IDP resettlement because they are residential zones that emerged from rural-urban migratory waves decades ago, but, contrary to Castilla, IPZ initiated as spontaneous marginal settlements that have been consolidated over time. The IPZs have produced permanent spatial configurations in the territory (i.e., paved streets, residential block shapes) and have established socio-spatial multicultural practices and structures (i.e., public spaces for celebrations, memorials). Our findings show that Downtown, Laureles, Castilla and IPZ formal areas provide opportunities to integrate new IDP communities. However, the most promising results, at least in a shorter period, seems to be in both Castilla and IPZ neighborhoods due to closer similarity of socio-spatial and cultural backgrounds to them. All of these neighborhoods have suitable infrastructure to support IDPs, such as health centers, schools, nurseries, recreational amenities, etc. In this sense, these neighborhoods allow for a smooth acculturation process and therefore offer more opportunities for a successful integration within the larger society.

The strength of our methodology lies in the integration of quantitative geospatial analysis with qualitative insights from participatory mapping, which ensures both accuracy and contextual relevance. Participatory mapping, in particular, plays a crucial role in validating the automated characterization of urban morphology derived from geospatial datasets. By involving not only experts but also IDP community leaders, we capture a more nuanced understanding of the socio-spatial dynamics in displaced communities. This combined approach mitigates the risk of perpetuating ineffective, top-down housing policies that fail to account for local realities. Instead, it provides a comprehensive framework for local authorities to generate more tailored, context-specific housing solutions. In this way, our methodology equips decision-makers with the necessary tools to address the unique challenges of displacement and resettlement more effectively.

## Acculturational housing policies as instrument to measure integration

Despite the diverse sociocultural-spatial backgrounds that IDPs bring to the cities in Colombia, the institutional response to their housing needs remains a one-size-fits-all. Previous works in developed countries have shown that synergies between geography and cultural backgrounds are important for refugee integration to the larger society (Bansak et al., 2018). Furthermore, Sam and Berry (2010) argue that neglecting the cultural backgrounds (acculturation) of merging groups to the larger society, increases their marginalization, as evidenced in our case study in Medellin. Thus, standardized housing solutions cannot embrace different socio-spatial cultural backgrounds, rather they disrupt the socio-spatial attributes coined by Lefebvre in his *spatial triad* conceptualization. Therefore, considering the economic and political limitations in Colombia to attend the humanitarian crisis of forced displacement, we propose three scenarios to improve housing solutions for IDPs, assessing the different levels of acculturation impact.

It is important to note that the content of this section presents recommendations rather than results. While Sect. 3 details our findings based on both qualitative and quantitative data, the three scenarios proposed here (marginal settlements improvement, new adaptive habitat solutions, and integrative housing solutions) are based on qualitative insights. These recommendations stem from our understanding that while quantitative methods are essential for analyzing urban morphology, addressing the housing needs of IDPs requires a qualitative approach that accounts for the socio-cultural complexities and historical failures of purely quantitative strategies. Therefore, the focus here is on proposing solutions that emphasize the lived experiences of displaced communities and their integration into housing policy.

### Marginal settlements improvement

**Fig. 9:**
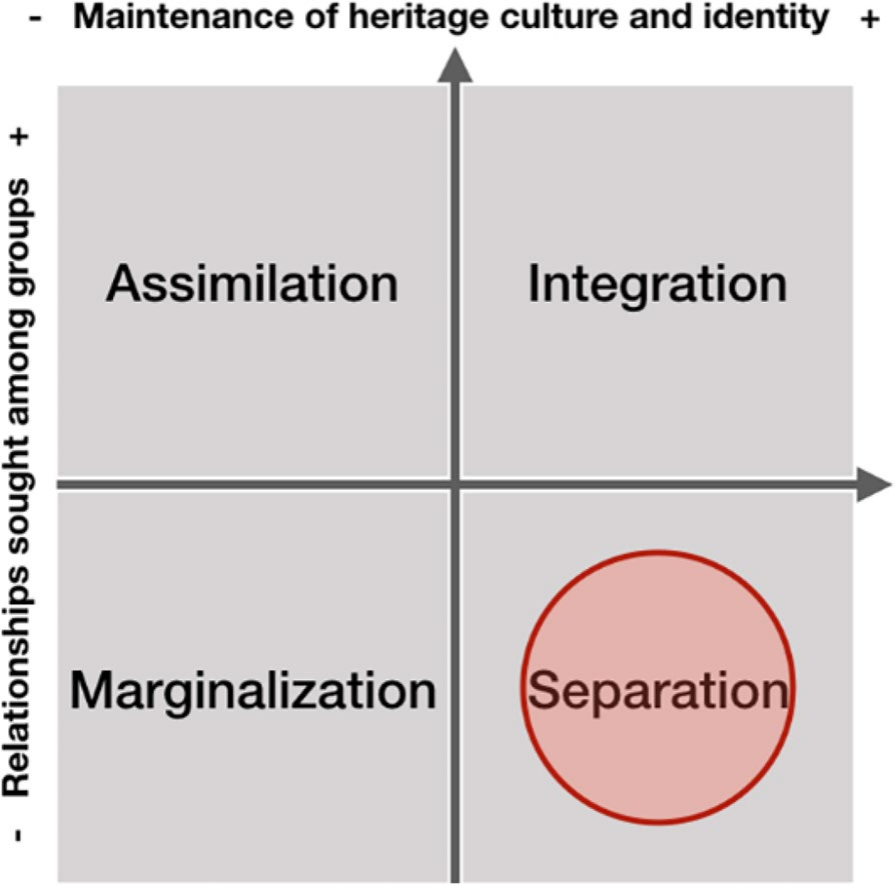
Acculturation scheme for settlement improvement. Attitudes of ethnocultural groups towards acculturation strategies in the larger society (Sam & Berry, 2010). The red circle (by authors) locates this solution within Sam and Berry’s acculturation scheme

The urban morphology metrics reveal settlements’ particular spatial characteristics. However, prior to any settlement improvement fieldwork and participatory methods (i.e., social mapping) would be necessary to identify and preserve subtle physical features related to the population’s cultural backgrounds. This would protect the existing sociocultural diversity and social tissues that indicate the ways how communities live and shape the social space, or in Lefebvrian terms, their spaces of representation. Additionally, spaces of collective significance shaped by social relations that go beyond the physical features (i.e., memorials, stairs with ramps) must be conserved because they represent socio-spatial realities and differences. However, while this approach can preserve socio-spatial cultural identity, it does not guarantee integration (rather, it promotes separation Fig. 9) with the larger society given that these communities often isolate themselves from daily interactions with members of other communities, especially due to discrimination.

### New adaptive habitat solutions

Before designing social housing developments, the similarity matrix as an instrumental tool, could provide benefits for decision making to local authorities and stakeholders. The diverse socio-spatial and cultural backgrounds of IDPs make the efficacy of homogeneous housing solutions unworkable. Thus, the results of the similarity matrix can be used to identify and group IDPs with compatible socio-spatial backgrounds even though if they have different origins. The heterogeneous spatial practices observed in IDP’s settlements suggest that new housing solutions should be the result of a participatory design during the planning process. They must have an adaptive architectural structure able for IDPs to dwell in multicultural communities. Therefore, while certain basic construction elements, such as building structure, water, and sanitation facilities, can be fixed, other components of the dwelling should be adapted to meet the individual needs of each family, such as the number and dimensions of rooms. Similarly, providing spaces for local commerce is a feature necessary for the economic survival of many IDP families, as observed in the visited marginal settlements. Hence, we posit that a democratic housing process and the cohabitation of similar cultural backgrounds could help to decrease the acculturative stress of IDPs that is generated by violent displacement. Although, this is a concept that requires further research.

**Fig. 10:**
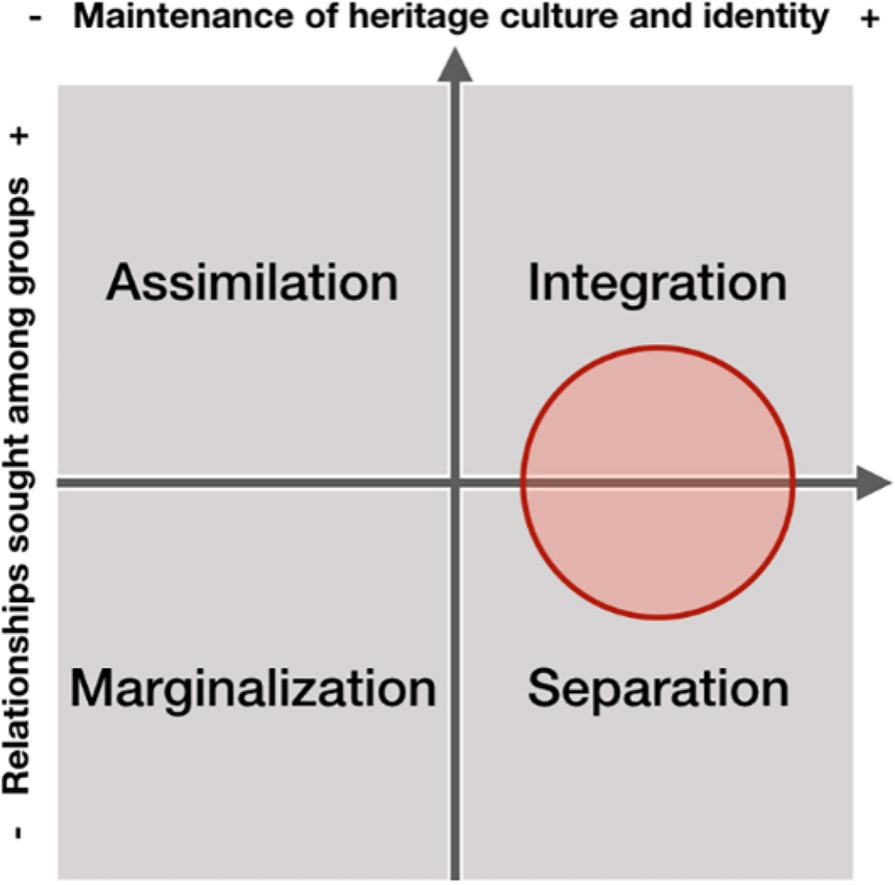
Acculturation scheme for new adaptive habitat solutions. Attitudes of ethnocultural groups towards acculturation strategies in the larger society by Sam and Berry (2010). The red circle (by authors) locates this solution within Sam and Berry’s acculturation scheme

Location is critical for new housing solutions for IDPs. Even though new adaptive housing solutions would improve living conditions (i.e., mitigation of risks to disasters) and change the way space is perceived by IDPs (i.e., precarity), their location on the margins of the city will endure cultural separation. Nevertheless, Fig. 10 illustrates how this approach, encouraging the coexistence of IDPs from different geographical origins but with compatible socio-spatial cultural backgrounds, could achieve an improvement in the integration dimension in comparison to the marginal settlements improvement.

### Integrative housing solutions

The triangulation of socio-spatial and acculturation analyses with the metrics assessment indicates that the integration of IDPs with the existing city is possible. Integrative housing solutions would offer dwelling opportunities (new or adapted) in already consolidated residential areas of the city due to the similarities with some marginal settlements. Hence, an interdisciplinary and close institutional commitment by relevant stakeholders (i.e., planning offices) is necessary for a successful outcome. In this scenario, Lefebvre’s representation of space concept, i.e., how space is conceived by planners, architects, technicians, plays a dominant role due to its operational complexity. Integrating IDP communities into the existing socio-spatial tissues of the city would eliminate the side effects of standardized housing solutions, and thereby decrease territorial marginalization.

**Fig. 11:**
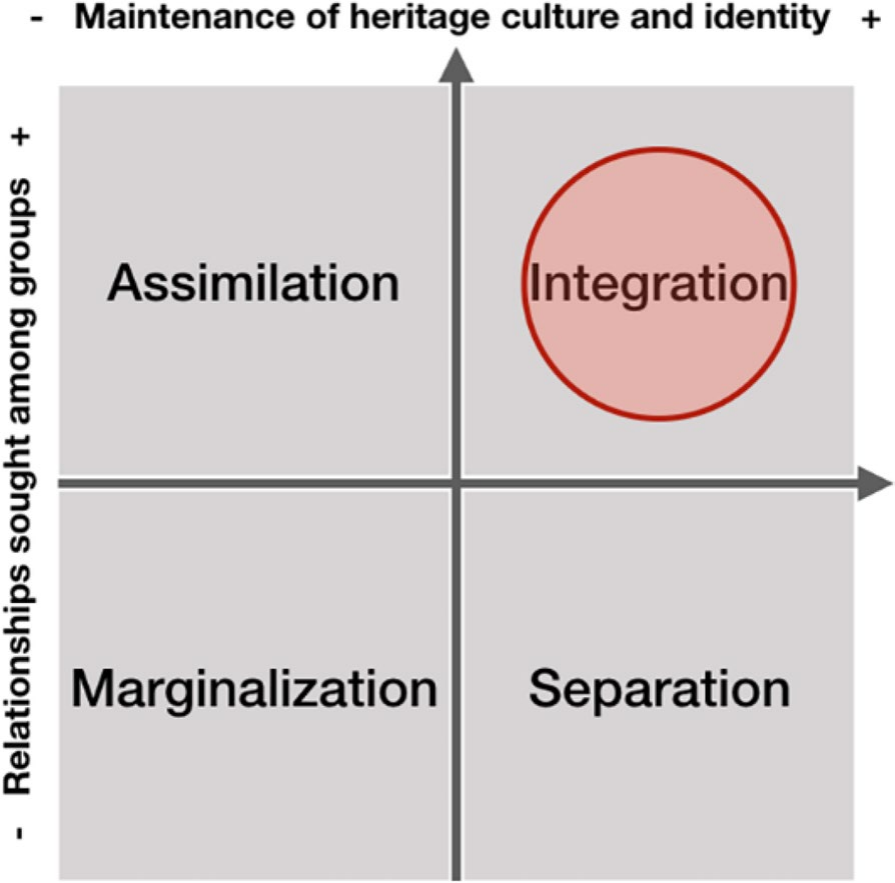
Acculturation scheme for integrative housing solutions. Attitudes of ethnocultural groups toward acculturation strategies in the larger society by Sam and Berry (2010). The red circle (by authors) locates this solution within Sam and Berry’s acculturation scheme

Many consolidated neighborhoods have opportunities to increase urban density, decrease dramatic urban growth and offer better living conditions for IDP communities. These neighborhoods usually have social services and access to public transportation, features that would stimulate city compactness and sociocultural integration while also saving economic resources when building new infrastructure. To make the integrative housing solutions more effective for IDP communities, a closer similarity analysis is necessary to determine the most compatible areas more precisely based on their socio-spatial and sociocultural backgrounds. Figure 11 illustrates the integration process of IDPs in the way the acculturative stress could be reduced through: (a) providing the opportunity to maintain their original culture and (b) encouraging daily life interactions with larger social networks.

### Long-term impact and sustainability

Within the uncertain and rapid nature of the forced displacement and resettlement process of IDPs in Colombia, driven by internal armed conflicts and constrained by the limited resources of local governments, we acknowledge the limitations of the proposed scenarios. However, the recommendations outlined in this paper do not call for significant additional investments but rather advocate for a more efficient allocation of existing or earmarked funds for housing IDPs. These suggestions are particularly relevant for Colombian stakeholders such as the Ministry of Housing (national level), ISVIMED (city level), and URAVID. By implementing these recommendations, it is possible to counteract the marginalization and socio-spatial injustices that contribute to violence, as discussed in the paper.

In the long term, the positive impact of these policies will be the reduction of social injustice, marginalization, and violence. This aligns with the principles of the Earth Charter, which calls for “a sustainable global society founded on respect for nature, universal human rights, economic justice, and a culture of peace.” A fairer and less violent city is a critical step towards achieving sustainability in both social and environmental dimensions.

### Best practices for applying these results

Based on the interdisciplinary methodology and findings presented in this study, we have identified several best practices that can guide the application of our results in the development of housing solutions for displaced populations. These practices aim to enhance the effectiveness and sustainability of resettlement efforts by integrating both socio-spatial and cultural factors, ensuring that housing solutions are not only technically sound but also socially and economically viable for the communities they serve.

#### Engagement and inclusion

Including IDPs in the data collection and decision-making processes is essential for designing accurate and effective housing solutions. The participatory approach allows IDPs to share their lived experiences and perceptions, offering insights that scientific and technical analysis alone cannot capture. Successful resettlement projects, such as the Juan Bobo Social Housing project in Medellín, demonstrate the importance of involving the community at every stage. This engagement not only helps build trust between the community and government but also legitimizes the voices of IDPs in the decision-making process, ensuring that housing solutions are culturally sensitive and contextually appropriate (Calderon, Slava, and Mejia, 2020).

#### Integration of socio-scientific and vernacular knowledge

Techno-scientific approaches alone are insufficient to address the complex socio-spatial needs of displaced populations. Combining quantitative data with qualitative insights (merging scientific and vernacular knowledge) enables a more holistic response to the displacement crisis. This hybrid approach ensures that housing solutions account for local socioeconomic conditions, cultural practices, and the specific needs of IDPs. For example, many IDP families in Medellín have returned to risk-prone areas after being relocated to high-rise buildings on the city’s outskirts due to the socioeconomic and cultural disconnects caused by the inappropriate relocation (e.g., isolation from informal work opportunities and social networks). Integrating these factors into housing management strategies can help prevent such setbacks and optimize public resources.

#### Culturally sensitive resettlement practices

IDP housing solutions should consider the cultural and economic practices of the displaced communities. Standardized housing often exacerbates marginalization by ignoring cultural factors such as communal living, informal economic activities, and spatial practices. Local authorities should adopt more flexible and adaptive housing models, allowing for customization based on cultural and economic needs. Participatory design processes, where IDPs actively contribute to the planning and design of their homes, can significantly reduce acculturative stress and foster long-term integration into the host community.

#### Proximity to livelihoods and social networks

Housing solutions should prioritize proximity to economic opportunities and social networks, particularly for informal workers. This is especially relevant for IDP communities, where many depend on local, informal economies for their livelihoods. Relocation to peripheral areas without adequate infrastructure or access to work can lead to economic hardship and social isolation. Ensuring that new housing developments are located near transportation hubs, markets, and employment centers will promote smoother transitions and more sustainable integration.

#### Capacity building for local governments

The implementation of socio-spatial analysis tools, such as those used in this study, equips local governments with the necessary analytical and operational capabilities to understand the dynamics of displacement and resettlement. By institutionalizing these tools and methods, governments can make more informed decisions, respond more rapidly to the needs of displaced populations, and plan for long-term integration. Additionally, local authorities should invest in training programs that empower public servants to engage with participatory methodologies and integrate them with technical planning.

#### Adapting housing to geographical and environmental conditions

As highlighted by the urban morphology analysis, informal settlements often adapt to the existing topographical conditions with innovative, locally developed solutions. New housing projects should consider these adaptations rather than imposing uniform designs that might not suit the geographical features of the resettlement areas. Flexible architectural designs that allow for topographical adaptations can prevent future risks and enhance the sustainability of housing projects.

#### Monitoring and feedback mechanisms

Post-resettlement monitoring and feedback loops should be established to continuously assess the well-being of resettled communities, and the effectiveness of the housing solutions provided. This would allow for adjustments and improvements to housing policies and practices, ensuring that they remain responsive to the evolving needs of IDP populations.

## Conclusions

We analyzed the physical appearance of different urban settlements in Medellin using three sequential methodological tools, namely, analytical, operational, and measurement. As an analytical tool, we used the spatial triad of Lefebvre for a better understanding of the data gathered through participatory mapping. Once identified IDPs’ settlements, as an operational tool, we used geospatial analysis to characterize the physical appearance of the settlements and perform a statistical correlation analysis to measure the similarities among the different settlement classes. Finally, we used a measuring tool, the acculturation chart proposed by (Sam & Berry, 2010), to estimate the level of marginalization that three proposed scenarios could provide to the IDP communities. The combination of quantitative and qualitative research methods proved useful in this work, as it helped to establish relevant knowledge specific to forced displacement resettlement contexts.

The results indicate that each marginal settlement resulting from the resettlement of IDPs has its own territorial signature that is intrinsically related to their inhabitants’ socio-spatial backgrounds. However, despite their socio-spatial-cultural diversity the institutional response for new housing is a one-size-fts-all solution that increases the marginalization and acculturative stress of the IDP. Being aware of the complexity and magnitude of the marginalization problem, and that marginalization is inversely proportional to integration (see Sam & Berry, 2010 and Figs. 7, 8 and 9), we argue that improvements to dwellings for IDPs could be achieved through the following three proposed approaches:

- Before improving current housing, authorities should analyze these two socio-spatial aspects of the settlements based on Lefevbre’s spatial triad: how the social space is perceived and lived. This would allow identification of spaces of collective and/or individual significance; social relations with the territory; habits; movement patterns; the human body in relation to the built environment; important symbols and artifacts; etc. Regardless, however, integration with the existing city is not guaranteed.
- New dwelling projects for IDPs should engage in adaptive habitat solutions. Through acculturation analysis, we believe that the acculturative stress in which IDPs have been immersed could be counteracted by grouping communities with similar socio-spatial backgrounds, even though they are not from the same place of origin. However, to overcome marginalization, separation from the larger society would still prevail.
- The combination of socio-spatial and acculturation studies with the results of the analysis of morphology metrics indicates that the integration between IDPs and the existing city could be possible. The city could be a recipient of the displaced population in consolidated, nonmarginal residential areas, which could result in the integration of displaced communities within the larger society and, thus, more opportunities and a more dignified life for IDP.

We contend that taking these aspects into account is a way to minimize the secondary effects of standardized housing solutions by promoting more integrated communities, decreasing acculturative stress, and thereby reducing violence. However, further local research is necessary from the perspectives of geospatial and socio-spatial theories to elaborate customized solutions that would facilitate gains, such as a better use of public funds, and mitigate ill practices, such as corruption and clientelism. We recognize the economic impact that the neoliberal policies of the Global South cities have on social housing projects where dwelling is considered a commodity rather than a human need. Therefore, this paper seeks to open debates regarding the possible ways in which alternative economic operational solutions could be designed with multiple stakeholders, including the private sector, to achieve more just and sustainable cities.

## Data Availability

Data used in this work will be available at https://github.com/Risegroup/UrbanFootprint once the paper is accepted.

## Abbreviations

IDP: Internally displaced people
CNMH: Centro Nacional de Memoria Histórica (National Center of Historical Memory)
UN: United Nations
DEM: Digital Elevation Model
WGS: World Geodetic System
EPSG: European Petroleum Survey Group
VHR: Very high spatial resolution
NDVI: Normalized difference vegetation index
NDBI: Normalized difference built-up index
